# Taking perspective the next time around. Commentary on: “Perceived perspective taking: when others walk in our shoes”

**DOI:** 10.3389/fpsyg.2015.00434

**Published:** 2015-04-10

**Authors:** Nathan N. Cheek

**Affiliations:** Department of Psychology, Swarthmore CollegeSwarthmore, PA, USA

**Keywords:** perspective taking, self-other overlap, egocentrism, social judgment, self-concept

The processes and consequences of perspective taking have attracted a substantial amount of research interest in recent years (e.g., Epley et al., 2004; Heller et al., 2008; Brown-Schmidt, 2009), and several studies have explored the many benefits of walking in another's shoes. For example, taking the perspective of someone else increases liking, prosocial behavior, and self-other overlap (for a review, see Galinsky et al., 2005). In an innovative series of studies, Goldstein et al. (2014) recently examined perspective taking from the other side, investigating the effects of *perceived perspective taking*—the feeling that others have walked in one's shoes.

Goldstein et al. (2014) demonstrated that perceived perspective taking has many of the same benefits as perspective taking: it similarly increases empathy, liking, prosocial behavior, and, most important to this commentary, self-other overlap. Self-other overlap describes the inclusion of others in the self-concept, resulting in an heightened sense of self-other similarity and closeness (e.g., Aron et al., 1991, 1992). When people take the perspective of someone else, the resulting increased self-other overlap helps reduce stereotyping (e.g., Galinsky et al., 2005; Wang et al., 2014). In Goldstein et al.'s studies, the increase in self-other overlap partially explained the increase in liking as a result of perceived perspective taking. Goldstein et al. concluded that perceived perspective taking has many positive benefits, and even suggested that it may increase the likelihood of reciprocal perspective taking (i.e., walking in the shoes of the person who took one's perspective) in the future.

Although I agree that both taking the perspective of others and perceiving that others have taken one's own perspective can have many positive outcomes, the relation between both processes and future perspective taking may not, in fact, be as positive as it appears at first glance. Indeed, I propose that the increased self-other overlap caused by both perspective taking and perceived perspective taking may, ironically, undermine future attempts at perspective taking, because a greater degree of self-other overlap actually impairs attempts to walk in other people's shoes (see Figure 1).

**Figure 1:**
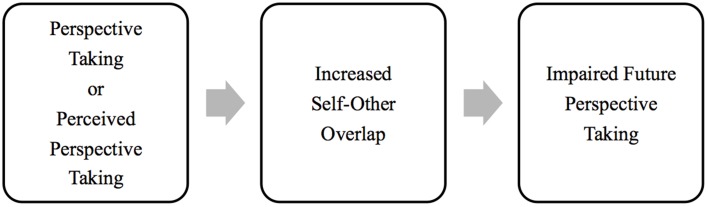
**Proposed effect of increased self-other overlap on future perspective taking**. Perspective taking and perceived perspective taking both increase self-other overlap (i.e., inclusion of the other in the self; Galinsky et al., 2005; Goldstein et al., 2014). This increase then impairs future perspective taking by causing people to overestimate the transparency of their thoughts and feelings to others, which causes them to behave more egocentrically (Vorauer and Cameron, 2002; Savitsky et al., 2011).

Successful perspective taking first requires that people differentiate the self from others, thereby recognizing that their thoughts and feelings are not necessarily shared by those around them (e.g., Apperly, 2010). As a result, significant self-other overlap may hinder perspective taking because people feel so connected with and close to others that they overestimate the transparency of their private inclinations, beliefs, and feelings. In other words, when attempting to take the perspective of others who have been included in the self, people often fail to appreciate that, despite the perception of closeness, others do not actually have full access to their perspective.

Vorauer and Cameron (2002) first explored this phenomenon in a series of studies on the effect of horizontal collectivism (i.e., attention to interdependence and equality and the feeling of being similar to others; Singelis et al., 1995) on people's perceptions of the transparency of their thoughts. Vorauer and Cameron found that people higher in horizontal collectivism believed that their thoughts and feelings were more accessible or obvious to close others. Moreover, this relation was mediated by self-other overlap, such that horizontal collectivism appeared to predict the inclusion of close others in the self, which then led people to overestimate the transparency of their perspective to others.

In a related study, Savitsky et al. (2011) introduced what they called the *closeness-communication bias*, which describes the tendency of people to be more egocentric when communicating with friends and other close others than when communicating with strangers. In one study, for example, participants engaged in a communication task with either a friend or a stranger. Participants sat on one side of a set of cubbies containing several items, and the friend or stranger sat on the opposite side and played the role of director, instructing participants to pick up target objects.

The key test of perspective taking was whether participants considered objects in their privileged ground (i.e., objects they could see but that were hidden from the friend or stranger giving directions). In one trial, for example, participants could see both a computer mouse and a stuffed toy mouse, whereas the director could only see the computer mouse. To measure participants' perspective taking, Savitsky et al. (2011) recorded participants' eye movements when the director instructed them to “pick up the mouse,” with fixations on an object representing consideration of it as a possible referent. Successful perspective taking would exclude the toy mouse from consideration, because participants knew that the director did not know it was there. Somewhat surprisingly, participants considered the toy mouse as a referent—that is, interpreted the instruction egocentrically—more often when the director was a friend than when the director was a stranger. Thus, participants were more successful at taking the perspective of another when they were interacting with a stranger than when they were interacting with a close friend.

In sum, a greater merging of the self and others appears, at least in some circumstances, to undermine people's ability to successfully take others' perspectives, largely because they fail to appreciate how different their perspective is from that of the people they include in their self-concept. Accordingly, that both taking perspective and perceived perspective taking increase self-other overlap suggests that they may actually impair, rather than improve, future attempts at perspective taking. Importantly, I do not intend to suggest that there are not many positive consequences of both processes—certainly, substantial research has documented many benefits (e.g., Galinsky et al., 2005). Nonetheless, to understand how people navigate the divide between what they know and what others know, it is also important to acknowledge the potential shortcomings of future perspective taking that result from self-other merging. For instance, perspective taking can improve performance in negotiations (Galinsky et al., 2008), but self-other merging may undermine perspective taking in future negotiations with the same opposing party. Walking in another's shoes is a worthwhile and fruitful endeavor, but one may find that the shoes do not fit as well the next time around.

## Conflict of interest statement

The author declares that the research was conducted in the absence of any commercial or financial relationships that could be construed as a potential conflict of interest.
